# A Molecular and Whole Body Insight of the Mechanisms Surrounding Glucose Disposal and Insulin Resistance with Hypoxic Treatment in Skeletal Muscle

**DOI:** 10.1155/2016/6934937

**Published:** 2016-05-05

**Authors:** R. W. A. Mackenzie, P. Watt

**Affiliations:** ^1^Department of Life Science, Whitelands College, University of Roehampton, Holybourne Avenue, London SW15 4DJ, UK; ^2^University of Brighton, Hillbrow, Denton Road, Eastbourne BN20 7SP, UK

## Abstract

Although the mechanisms are largely unidentified, the chronic or intermittent hypoxic patterns occurring with respiratory diseases, such as chronic pulmonary disease or obstructive sleep apnea (OSA) and obesity, are commonly associated with glucose intolerance. Indeed, hypoxia has been widely implicated in the development of insulin resistance either via the direct action on insulin receptor substrate (IRS) and protein kinase B (PKB/Akt) or indirectly through adipose tissue expansion and systemic inflammation. Yet hypoxia is also known to encourage glucose transport using insulin-dependent mechanisms, largely reliant on the metabolic master switch, 5′ AMP-activated protein kinase (AMPK). In addition, hypoxic exposure has been shown to improve glucose control in type 2 diabetics. The literature surrounding hypoxia-induced changes to glycemic control appears to be confusing and conflicting. How is it that the same stress can seemingly cause insulin resistance while increasing glucose uptake? There is little doubt that acute hypoxia increases glucose metabolism in skeletal muscle and does so using the same pathway as muscle contraction. The purpose of this review paper is to provide an insight into the mechanisms underpinning the observed effects and to open up discussions around the conflicting data surrounding hypoxia and glucose control.

## 1. Introduction

Type 2 diabetes is a metabolic disease categorized, primarily, by reduced insulin sensitivity, *β*-cell dysfunction, and elevated hepatic glucose production [1]. Insulin resistance is widely accepted as the starting point for the progression from glucose intolerance to overt type 2 diabetes. Therefore understanding the underlining mechanisms of insulin resistance pathophysiology is of great importance to the development of novel and effective treatments.

Peripheral insulin resistance represents a decrease in insulin-dependent glucose transport in insulin responsive tissues [2], which can be the product of defects at both the insulin receptor and/or postreceptor signaling [3]. Inflammation [4–7], hyperglycemia [8, 9], hyperinsulinemia [10, 11], hyperlipidaemia [12, 13], and hypoxia [14–16] have all been linked to the development of insulin resistance and type 2 diabetes. Indeed, hypoxia has been widely implicated in the development of insulin resistance either via the direct action on insulin receptor substrate (IRS) [17] and protein kinase B (PKB; also known as Akt) [18, 19] or indirectly through adipose tissue expansion [20] and systemic inflammation [21, 22]. However, we demonstrated that acute hypoxic exposure increases two-compartment models of insulin sensitivity (*S*_*I*_^2*∗*^) in human type 2 diabetics [23] with Lecoultre et al. [24] showing that ten nights of moderate hypoxic exposure improved insulin sensitivity in obese males, as measured by the 2-step hyperinsulinemic-euglycemic clamp method. This data highlights some of the controversy over the role hypoxia plays in glucose control and metabolism. Interestingly, the same work showed that muscle expression of Akt and IRS1 was not affected by the hypoxic treatment [24].

The purpose of this review is to consider the literature while providing roles for hypoxia in causing insulin resistance and glucose intolerance. Furthermore, this review will discuss hypoxia's apparent dual ability to increase glucose transport activity acutely in skeletal muscle, using a pathway independent of insulin, and dissect why hypoxia is also implicated in insulin resistance. Providing a greater understanding of the metabolic responses to hypoxia has genuine clinical relevance and may open up future therapeutic methods in the treatments of glucose intolerance and type 2 diabetes.

## 2. The Evidence Surrounding Hypoxic-Induced Insulin Resistance

The observation that insulin resistance and glucose intolerance are positively correlated with hypoxia originates from studies by Strohl et al. in 1994 [25]. These authors suggested that sleep apnea was independently associated with body mass index (BMI) and insulin dysregulation [25]. The chronic or intermittent hypoxia patterns occurring with respiratory diseases, such as chronic pulmonary disease or obstructive sleep apnea (OSA), are commonly cited as potential causes of glucose intolerance [26–28], an early indication of a disruption in normal glycemic control. The link between respiratory diseases and insulin resistance is complex. Oltmanns et al. [16] clearly showed that glucose infusion rates are reduced in response to a 30-minute period of sustained hypoxia (oxygen saturations levels ~75%). The authors attribute this to a sympathoadrenal-induced epinephrine release, resulting in increased hepatic glucose production and a reduction in glucose disposal at insulin sensitive peripheral tissue [16]. In addition, epinephrine has been shown to inhibit insulin-stimulated glucose uptake in rat skeletal muscle by reducing glucose phosphorylation [29]. The hypoxic stimulus used in Oltmanns et al. [16] work is likely to have increased hepatic glucose appearance [30] in an attempt to offset a change in peripheral tissue fuel utilization towards glucose metabolism. The consequence would therefore be a reduction in dextrose infusion rates [16] and a decrease in insulin mediated glucose uptake [29]. Indeed, epinephrine causes GLUT-4 translocation yet inhibits insulin mediated glucose disposal skeletal muscle [31]. Thus the responses described might reflect a transient change in metabolic processes and not chronic changes in insulin resistance.

Brooks et al. showed that insulin concentrations were elevated in healthy individuals upon arrival to high altitude (4,300 metres) [32], suggesting that hypoxia may cause glucose intolerance or at the very least disrupt glucose metabolism. However this is hard to conclude without access to c-peptide measures, which were not presented in this work [32]. Furthermore, the rise in blood insulin values may actually be a product of reduced insulin action rather than decreased glucose uptake. In more recent work, Louis and Punjabi [33] demonstrated a reduction in one-compartment models of insulin sensitivity (*S*_*I*_) and insulin secretion in response to 5 hours of intermittent hypoxia, a treatment used to replicate OSA. In this study it was suggested that increased sympathetic nervous activity, in response to intermittent hypoxia, decreased glycogenesis, increased glycolysis, and diminished the ability of glucose to stimulate its own uptake and disposal [33, 34], which is a very confusing message. Yet if we unpick this, it may provide some clarity to the underlining metabolic response to hypoxia. We suggest that hypoxia stimulates a stress pathway for glycolysis while blocking insulin facilitated glucose uptake, providing a reason for the reduction in glycogenesis seen in the work of Louis and Punjabi [33]. However, this would not explain the decrease in the ability of glucose to stimulate its own transport, measured in Louis and Punjabi [33] work as glucose effectiveness (*S*_*G*_). However, *S*_*G*_ is a mixed parameter that measures the ability of glucose to affect its own transport by mass action at basal insulin concentrations and is therefore dependent, to an extent, on insulin.

In other reports insulin resistance increases with hypoxia in genetically leptin deficient obese mice [15], respiratory conditions [35], and healthy humans [14, 36]. However, a closer look at the underlining data from these reports is informative and helps unpick more detail around the response of different models of hypoxia. For example, the increase in insulin resistance noted in the Polotsky et al. [15] study, which used obese, leptin deficient mice, was completely abolished by acute leptin replacement. Leptin, an adipose tissue peptide hormone, interacts with skeletal muscle [37], increasing fatty acid oxidation, and reduces intramuscular stores of triglycerides [38] while improving insulin action [39]. There is evidence to show that a decrease in secondary lipid products, ceramide, diglyceride, and long-chain fatty acyl CoA can reduce the inhibitor effect of fats on Akt mediated insulin signaling in skeletal muscle (reviewed [40]).

Using the typically regarded “gold standard” assessment of glucose tolerance (euglycemic-hyperinsulinemic clamp), Larsen et al. [41] found that insulin sensitivity decreased significantly in response to 2 days of altitude exposure (4559 m; ~12% O_2_) with a reduction in glucose infusion rates to achieve euglycemia, from 9.8 (1.1) to 4.5 (0.6) mg·kg^−1^·min^−1^ (*P* < 0.05). The same work did, however, show improvements in insulin action with altitude acclimatisation (7-day exposure) [41] suggesting a haemostatic balance between insulin secretion, insulin action, and glucose disposal rates.

From a cellular mechanistic point of view, hypoxia seems to induce insulin resistance in insulin sensitive tissue through the suppression of total Akt during basal conditions and with IGF-1 stimulation in C2C12 skeletal muscle cells [42]. Low oxygen treatment of C2C12 cells* in vitro* inhibits the PI3-kinase/Akt pathway by reducing IGF-I receptor (IGF-IR) sensitivity to growth factors [19], suggesting that hypoxia may interfere directly with key signaling transduction pathways in skeletal muscle. In addition, the same work showed that pGSK3*α* ^S21^, pGSK3*β* ^S9^, total GSK, pAkt^t308^, pAkt^s473^, and total Akt were all reduced following 48 hours of differentiation in C2C12 while IRS-1 and IRS-2 were unchanged under severe hypoxic treatment (O_2_~ 0.5%) [19]. This again suggests that hypoxia alters insulin signaling at a postreceptor-intracellular level and/or via an indirect action on IGF-1 receptor. All of this data taken together demonstrates that hypoxia, at least in* in vitro* models, has the ability to alter insulin signaling of IRS downstream. It is worth mentioning that the relevance of culture models to whole body physiological responses must be read with a degree of caution as the level of hypoxia (i.e., O_2_ ~ 0.5–5%) commonly used* in vitro* work is unlikely to be seen at the tissue level of skeletal muscle in humans subjected to whole body hypoxia (i.e., O_2_ ~ 12–15%). However, phosphorylation of Akt^s473^ and GSK-3*β* ^s9^, obtained from vastus lateralis using standard muscle biopsy techniques, was decreased in chronic obstructive pulmonary disease (COPD) patients presenting with hypoxemia (resting arterial PO_2_ = 57.0 (1.0) mmHg) [43]. This evidence suggests that chronic hypoxemia seen in disease conditions may be involved in the progression of insulin resistance. However, these findings were not supported in skeletal muscle extracted from C57BL/6J mice treated with 10% O_2_ for 4 weeks [44]. Interpretations from human work are also mixed with Etheridge et al. [45] showing no change in pAkt^s473^ during hypoxia while D'Hulst et al. showed that 11% inspired O_2_ reduced pAkt^s473^ [46]. The difference between these studies may be explained by the basal nutritional status of the subjects. Etheridge and colleagues [45] examined their subjects in a fasted state, whereas those in D'Hulst and colleagues study [46] consumed a meal 40 minutes prior to the start of the experimental trial.

Lastly, whole body hypoxic treatment seen in* in vivo* research is likely to affect a variety of tissue types, not just skeletal muscle. Obesity is characterized by adipose tissue expansion that results in pockets of localised tissue hypoxia in the most affected areas. In addition, there is evidence that localised hypoxia seen in adipose tissue may result in systemic metabolic dysfunction seen in different tissue types, further highlighting the complexity of the issue. For this reason the effects of hypoxia on fat tissue have been widely researched. In an attempt to isolate the effects of hypoxia on adipocytes, Regazzetti et al. [47] treated 3T3-L1 cells with 1% O_2_ and showed that Akt, pAS160 content, and glucose transport rates were all decreased under these conditions and that this stress further inhibited insulin signaling and glucose uptake in response to insulin treatment [47]. Thus the response of whole body metabolism to hypoxia may be a systemic condition. Nevertheless, hypoxia clearly alters metabolism and affects intracellular signaling of tissues which is likely to serve the goal of reducing energy consuming processes (i.e., glycogen formation and protein synthesis) and upregulate ATP producing (i.e., glycolysis) and cell survival mechanisms.

## 3. Hypoxia Stimulates Glucose Uptake Independent of the Actions of Insulin

**Figure 1:**
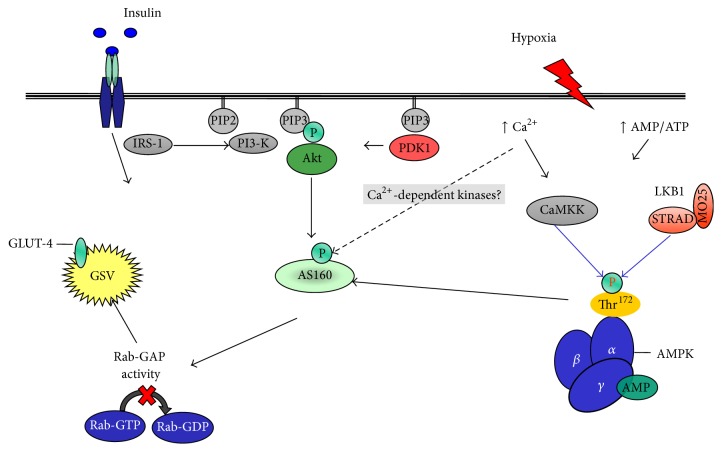
Insulin and contraction signaling pathways during GLUT-4 recruitment and translocation. Adapted from Mackenzie and Elliott [60]. IRS, insulin receptor substrate; PI3-K, class IA phosphatidylinositol 3-kinase; PIP2, phosphatidylinositol 4,5-bisphosphate; PIP3, phosphatidylinositol 3,4,5-trisphosphate; PDK1, phosphoinositide-dependent protein kinase-1; Akt, serine/threonine protein kinase; AS160, 160 kDa Akt substrate; GLUT-4, glucose transporter 4; GSV, GLUT-4 storage vesicle; Rab-GAP, Rab-GTPase-activating protein; Rab-GDP, guanosine-50-diphosphate-loaded Rab; Rab-GTP, guanosine-50-triphosphate-loaded Rab; CaMKK, Ca^2+^/calmodulin-dependent protein kinase kinase; LKB1, serine/threonine kinase 11; STRAD, putative kinase; MO25, mouse protein 25/scaffold protein; AMPK, 5′-monophosphate-activated protein kinase; Thr^172^, phosphorylated AMPK*α* at threonine 172; AMP, adenosine monophosphate; ATP, adenosine triphosphate; P, phosphorylated site.

Insulin and contractile activity stimulate glucose disposal in skeletal muscle using separate, independent signaling pathways [48] with insulin mediated via Akt-AS160 and contraction via AMPK-AS160. Hypoxia also activates glucose transport using the same signaling pathway as that of contractile activity [49] (Figure 1). Indeed, glucose transport has been shown to be additive when either hypoxia or contractile activity is coupled with insulin, whereas hypoxia and contractile activity are not [49, 50]. The ability of hypoxia to stimulate glucose disposal, independently of contractile activity, has been documented in both animal [49, 51] and* in vitro* work using isolated human muscle tissue [48, 50].

In 1958 Randle and Smith published data showing that hypoxia, induced via the chemical inhibition of oxidative metabolism, resulted in a loss of cellular potassium (K^+^), inhibition of active K^+^ uptake, coupled with stimulation of ATP-sensitive K^+^ channels, an increase in cellular ATP/AMP ratio, and ultimately an increase in extracellular K^+^ levels [52, 53]. These ion changes lead to membrane depolarisation, opening of voltage-gated Ca^2+^ channels, and an increase in SR Ca^2+^ release in a manner similar to muscle contraction. Hypoxia is known to reduce oxygen availability, inhibit mitochondrial respiration [54], and increase AMP : ATP ratio [55], resulting in increased cytolytic AMP availability and greater AMP binding capacity to the *γ* regulatory subunit, activation of AMPK [56], and stimulation of glucose transport [49].

It is clear that hypoxia (at least during the stress) encourages glucose uptake in skeletal muscle via AMPK and Ca^2+^-dependent mechanisms. Evidence shows that Ca^2+^ can activate glucose uptake in a calmodulin-dependent protein kinase (CaMKK)/AMPK-dependent manner [57] identical to mechanisms responsible for contraction induced glucose uptake in muscle [48, 49], although elevations in intracellular Ca^2+^ levels may also provoke AMPK-independent glucose transport as glucose transport activity is increased during subcontraction increases in muscle Ca^2+^ when stimulated with caffeine [56]. Activation of glucose uptake with hypoxia is facilitated by an increase in the activation of GLUT-1 preexisting in the cell membrane [54] while stimulating translocation of intracellular GLUT-1 and GLUT-4 to the sarcolemma [49, 54].

AMPK is essential for hypoxia-induced glucose transport [58]. Using AMPK*α*2 deficient rodents (Tg-KD1), Mu et al. [58] showed that glucose transport was completely blocked under hypoxia when compared to wild-type counterparts. Furthermore, the hypoxia-induced increase in membrane bound GLUT-4 content was reduced in the same Tg-KD1 mice [58]. These findings have been extended into human work, with Wadley et al. [59] showing that AMPK*α*2 activity and AMPK*α* Thr^172^ phosphorylation were significantly increased during exercise in hypoxia when compared to the same relative exercise intensity in normoxic conditions. The rate of glucose disappearance was also found to be significantly higher in the hypoxic trial, suggesting that hypoxia, when combined with exercise, has a greater effect on AMPK activity and glucose transport over exercise alone [59] and that hypoxia mediates glucose uptake via a pathway dependent (partly) on AMPK in humans.

Much of the work discussed above is in cell culture models or animal work, yet the findings from a whole body perspective seem to align well with this data. Using isotope methodology Brooks et al. [32] concluded that altitude acclimatisation (4300 m) increased glucose disappearance (*R*_*d*_) and metabolic clearance rates (MCR) during both exercise and resting states when compared to sea-level values. Interestingly, insulin concentrations were unchanged from prealtitude levels [32], suggesting an increase in contraction-stimulated glucose transport or improved insulin sensitivity at altitude. In support Johnson et al. [61] demonstrated that acute altitude exposure (2–40 hr) resulted in progressive hypoglycemia, which was attributed to increased glucose clearance and oxidation, which was confirmed by Cooper et al. [62]. Using an oral glucose tolerance test, Lee et al. [63] showed that high altitude exposure (3 days) significantly improved glucose tolerance in sea-level natives and in type 2 diabetics. Prior hypoxic exposure is also known to increase two-compartment models of insulin sensitivity [23] with acute intermittent hypoxia shown to improve glucose control in patients with type 2 diabetes [64].

A study by Forbes, in 1936, was one of the first to suggest that altitude could alter the manner in which glucose is handled by health sea-level residents [65]. This work showed that blood glucose clearance was increased during an OGTT administrated at high altitude. Following this work, research has not only confirmed Forbes [65] conclusions but looked to extend them by showing that long-term exposure to simulated or actual altitude results in (1) reduced fasting plasma glucose concentrations [66–69] and (2) elevated glucose clearance rates during an intravenous glucose load [66, 68].

A recent review concluded that long-term exposure to altitude results in improved glycemic control and lower prevalence of obesity and diabetes [70]. It is generally recognised that high altitude natives have a reduced prevalence of type 2 diabetes [67, 71, 72] while the same population also displays lower glucose concentrations (50.6 (3.7) mg/dL) compared to sea-level residents (73.4 (4.0) mg/dL) when monitored during ~12-hour period [73]. Despite having a high prevalence of obesity (BMI ≥ 30 kg/m^2^) rural Aymara natives (living at altitudes 2050–4250 m) are also known to be at a reduced risk of developing type 2 diabetes [72]. These authors attributed this finding to near normal insulin values (mean (SD); 9.3 (10.2) *µ*U/mL) and low levels of insulin resistance (HOMA_IR_ 1.8 (2.4)) [72].

Studies elsewhere have shown that long-term altitude exposure is linked with low glucose and insulin concentrations [74, 75]. Ge et al. [76] concluded that Tibetan natives exhibit genetic modification (namely,* PPARA*, encoding PPAR*α*) that increases glycolysis and decreases hepatic gluconeogenesis and free fatty acids. The authors further suggest that these adaptations may help to reduce diabetic and obesity risk [76]. Indirect evidence also shows inverse correlations between altitude natives and the risks of diabetes [77].

### 3.1. Insulin Resistance Seen with Hypoxia May Merely Reflect the Use of a Separate Preferential Pathway for Glucose Uptake

The literature surrounding hypoxic induced changes in glycemic control, insulin resistance, and type 2 diabetes may appear as confusing and conflicting. How is it that hypoxia can seemingly cause insulin resistance while at the same time stimulating glucose uptake? The answer may be attributed to the duration of stress (minutes versus days), the host (i.e., altitude natives versus obese type 2 diabetics), the nutritional status of the host, the model under investigation (cell culture versus whole body), the degree of adaptation to the conditions, and the measurement methods under use (hyperinsulinemic-euglycemic clamp during hypoxia), if we can put all of these matters to one side for the time being and remember that hypoxia is stress and that, under such conditions, respiring tissue seems to switch to a stress mediated pathway acting independently, in this context, to insulin. This has been demonstrated indirectly through the suppression of insulin action [14, 16] and insulin secretion with hypoxic treatment [78], while, at the same time, encouraging glucose uptake [32, 49]. We suggest that under hypoxic condition a preferential Ca^2+^/AMPK-dependent pathway may be upregulated to maintain ATP production (glycolysis) and reduce ATP consuming mechanisms (protein and glycogen synthesis). This is partly supported by the literature that shows that hypoxia and exercise stimulate glucose uptake via AMPK-AS160 [58, 79] while insulin acts through Akt-AS160 [80–82] mediated pathways. Comparisons with exercise are used, as this stimulus is known to activate glucose uptake using the same mechanisms associated with hypoxia.

Hypoxia has been shown, in cell lines, to impair IRS-1 [47], Akt, and PI3-kinase activity [19, 42], yet the same stress increases glucose uptake, intracellular Ca^2+^ levels, CaMKK, AMPK-AS160, and GLUT-4 muscle content. In support, whole body experimental work would suggest that hypoxia induces insulin resistance while also stimulating glucose disposal. This review will now try to explain these contrasting views while proposing a working hypothesis as to the role of hypoxia in glucose metabolism.

Firstly, the findings from whole body studies that hypoxia causes insulin resistance may be the product of increased insulin release combined with a decrease in insulin action at the site of insulin sensitive target tissue. Indeed, elevated circulating insulin concentration and perceived insulin resistance with hypoxic treatment [83] are a finding that has some support. In addition, hypoxia is known to encourage insulin synthesis and release by pancreatic *β*-cells [84], creating an acute or chronic physiological state resulting in blood insulin accumulation and perhaps perceived insulin resistance. This combined with decreased insulin-stimulated glucose disposal and insulin signaling is likely to result in such conclusions. Further, the introduction of exogenous insulin, which is implicit in the hyperinsulinemic-euglycemic clamp method, may further aggravate the problem. Hyperinsulinemia has been demonstrated to inhibit IRS-1/Akt activity* in vitro* [11] and cause insulin resistance* in vivo* [10, 17, 85, 86]. Thus the use of the two-step hyperinsulinemic-euglycemic clamp which delivers low insulin (20 mU·m^−2^·min^−1^ for 180 min) followed by high insulin dose (80 mU·m^−2^·min^−1^ for 120 min) [24] may be a better approach to assessing insulin sensitivity in insulin resistant/obese populations under additional environmental hypoxia. Importantly, Lecoultre et al. [24] work measured insulin sensitivity under normoxic conditions both before and after the ten-day treatment. Thus the clamp method here was employed following low oxygen treatment, rather than during hypoxic conditions.

Here we suggest that combining hypoxia with insulin accumulation, through its exogenous introduction and/or decreased insulin action, would indicate that the use of clamp methods is not necessarily an appropriate model for assessing insulin sensitivity under nonsteady state conditions such as exercise or hypoxia. Indeed, the introduction of exogenous insulin with the hyperinsulinemic-euglycemic clamp and a decrease in insulin signaling activity [17] due to heavier reliance on contraction-stimulated glucose uptake may merely reflect a shift towards a preferential pathway for glucose uptake resulting in plasma insulin accumulation.

There may also be some intracellular cross-talk between the two prominent regulatory pathways involved in controlling glucose uptake under hypoxic conditions which results in upregulation of AMPK-AS160 that coexists with a reduction in insulin mediated glucose transport. There is certainly some good evidence that hypoxia may cause insulin resistance; however it is proposed that this may be a product of a competition between pathways (i.e., a preferential use of the AMPK pathway under hypoxic stress) with a subsequent decrease in insulin signaling rather than insulin resistance* per se*. This is supported by the direct inhibition of insulin stimulated glucose transport with treatment of the calmodulin antagonist N-(6-aminohexyl)-5-chloro-1-naphthalenesulfonamide (W-7) [87]. Calmodulin (CaM) is a calcium-binding protein that modifies target proteins [88] such as AMPK [89]. In addition this Ca^2+^/calmodulin complex is considered to be involved in hypoxic induced glucose uptake [90] with AMPK activation increased with the overexpression of Ca^2+^/calmodulin-dependent protein kinase (CaMKK) [89]. In addition, the downregulation of CaMKK using RNA interference inhibits AMPK activity [89], implicating CaMKK in AMPK regulation and glucose uptake [48, 56, 91, 92].

**Figure 2:**
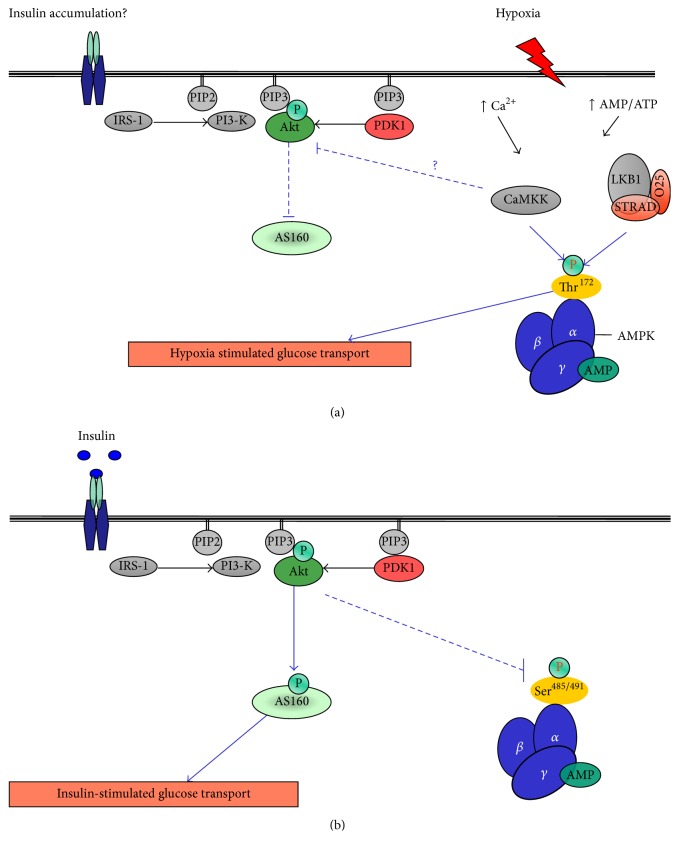
Proposed communication between hypoxia- and insulin-stimulated glucose transport mechanisms. (a) displays the proposed hypoxia-induced Ca^2+^/CaMKK inhibition on insulin signaling and insulin-stimulated glucose transport. Glucose transport is facilitated via an AMPK-dependent mechanism in response to hypoxia despite decreased Akt activity. (b) Glucose transport mechanisms in response to insulin. (b) also displays Akt mediated AMPK phosphorylation at Ser^485/491^ [93] resulting in AMPK inhibition.

Hypoxia increases intracellular free Ca^2+^ [49] and subsequently CaMKK [90] with the ensuing calcium signaling directly inhibiting insulin-stimulated glucose transport. The proposed mechanisms involved in this inhibition are detailed in Figure 2(a). Youn et al. [87] showed that the effects of W-7 treatment on glucose transport were additive with hypoxia and that the same treatment reduced insulin mediated glucose transport in skeletal muscle. Furthermore, a fivefold increase in insulin concentration was required to produce a half-maximal stimulation in glucose transport [87]. All of this data combined indicates that hypoxia-induced glucose transport is not hindered by W-7 treatment and that free intracellular Ca^2+^ may directly or indirectly inhibit insulin signaling. This data suggests that the upregulation of glucose uptake by hypoxia may result in insulin accumulation systemically, by the effect of reduced processing of insulin into insulin fragments by insulin sensitive tissues. The latter point is important as this may lead to the conclusion that hypoxia causes a reduction in insulin action and results in glucose intolerance due to plasma insulin accumulation.

Further evidence of the regulatory pathways that affect glucose transport work independently of each other, yet having the ability to communicate with one another, comes from experiments with 5-aminoimidazole-4-carboxamide ribonucleoside (AICAR), which activates AMPK while inhibiting insulin-stimulated glucose transport in 3T3-L1 adipocytes [94]. Incubating C2C12 skeletal muscle cells or rat extensor digitorum longus (EDL) muscle with insulin increases phosphorylation of AMPK at S485/491 [95]. Phosphorylation of AMPK at S485/491 directly inhibits AMPK activity. Indeed, insulin stimulation of Akt results in pAMPK^S485/491^, leading to a reduction in AMPK activity [93] (Figure 2(b)). This phosphorylation can prevent subsequent activation of AMPK^Th172^ by LKB1 and that pAMPK at S485/491 by insulin reduces the interaction between AMPK and LKB1 [96] suggesting that insulin inhibition of AMPK occurs upstream and that insulin, via the activation of Akt, may directly interfere with AMPK phosphorylation and activity [93], while presumably reducing AMPK-dependent glucose uptake. Collectively, these data again suggest that a potential cross-talk interplay between the Akt and AMPK pathways may exist and that the downregulation of insulin signaling by hypoxia reduces the Akt inhibitory effect on pAMPK^S485/491^.

## 4. Concluding Remarks

Evidence that hypoxia leads to insulin resistance has been widely published. There seems little doubt that acute hypoxia interferes with insulin signaling/action in skeletal muscle. Yet the same stress is also partnered with increased glucose uptake in a largely insulin independent manner.* In vivo* research presents data which is conflicting, some showing improvements in insulin sensitivity while others suggesting that hypoxia induces insulin resistance. At a cellular level, there seems to be less controversy. Hypoxia increases glucose uptake and activates Ca^2+^/AMPK mediated pathways in response to low oxygen tension in both cell culture models and* ex vivo* skeletal muscle. Yet, the same stress seems to decrease receptor and postreceptor activity of key insulin signaling intermediates. Firstly, we suggest that hypoxia does indeed downregulate insulin signaling, at least in skeletal muscle, and that a reduction in this pathway ultimately results in insulin accumulation and results in the misleading detection of insulin resistance, making the* in vivo* modelling of insulin sensitivity under hypoxic conditions difficult, particularly with the introduction of high physiological concentrations of exogenous insulin as associated with the hyperinsulinemic clamp approach. It seems clear that the two major pathways involved in glucose transport and metabolism in skeletal muscle, insulin- and contraction-dependent mechanisms, work separately and that upon the application of stress (i.e., hypoxia) cells shift towards a preferred AMPK-dependent mechanism and away from insulin. This is merely a working hypothesis but is not a new notion with Cartee et al. [49] and Azevedo et al. [50] clearly demonstrating that hypoxia activates the contraction-stimulated pathway to facilitate glucose transport and that this occurs independent of the actions of insulin.

It is important that we develop a better understanding of glucose transport mechanisms and the causes of insulin resistance as this has clear clinical applications. Furthermore, it may be that hypoxia has a part to play in the therapeutic treatment of type 2 diabetes rather than being implicated in its progression. Conclusions from* in vitro* work are important but limited in application. At a whole body level, many of the studies that demonstrate a link between insulin resistance and hypoxia have done so while measuring this parameter under low oxygen conditions, which, while being a valid approach in terms of external validity, makes the modelling of insulin sensitivity difficult. Thus developing new* in vivo* approaches to modelling insulin sensitivity, such as the two-step clamp method, may increase our understanding of the role hypoxia plays in glucose transport and glycemic control. Glucose effectiveness, as measured by the labelled intravenous glucose tolerance test (IVGTT), quantifies the ability of glucose to transport itself at basal insulin concentration. This technique also separates glucose control into measures of hepatic glucose production and disposition index and, as such, may provide a useful approach to assessing the true effects of hypoxia on insulin and hypoxic mediated glucose disposal.

The question of whether hypoxia causes insulin resistance, or not, is a complex one. At a cellular level, the evidence clearly shows that hypoxia increases glucose uptake and that this coexists with an inhibition of insulin signaling. The authors propose that under hypoxic conditions, at least acutely, glucose transport is increased using cellular pathways that operate independent of insulin thus given the impression of insulin resistance. Given the current research, the authors conclude that hypoxia may decrease insulin signaling but may not induce whole body insulin resistance.
